# Mechanical Performance of Copper-Tailings Concrete with Steel Fibers for Potential Underground Support Applications

**DOI:** 10.3390/ma19173794

**Published:** 2026-09-06

**Authors:** Cristopher Hernández, Belén Barraza, René Gómez, Krzysztof Skrzypkowski, Jerzy Stasica, Zbigniew Rak

**Affiliations:** 1 Department of Mining, Metallurgical and Materials Engineering, Universidad Técnica Federico Santa María, Santiago 8940897, Chile; 2 Laboratory of Sustainable Processes, Department of Mining, Metallurgical and Materials Engineering, Universidad Técnica Federico Santa María, Santiago 8940897, Chile; 3 Faculty of Engineering, Universidad de Concepción, Concepción 4030000, Chile; 4 Faculty of Civil Engineering and Resource, AGH University of Krakow, Mickiewicza 30 Av., 30-059 Krakow, Poland

**Keywords:** circular economy, copper tailings, fiber-reinforced concrete, underground mining

## Abstract

The increasing accumulation of mine tailings has motivated the development of sustainable strategies for their reuse within the framework of the circular economy. This study evaluates the mechanical performance of four laboratory-cast concrete mixtures: a reference mixture, a mixture in which copper tailings were incorporated at 8% of the base mixture mass to replace an equivalent mass of natural coarse sand, and two tailings-based mixtures reinforced with 0.4% and 1.2% steel fibers. Their compressive strength and elastic modulus were evaluated after 7, 14, 28, and 100 days of curing to assess the mechanical performance of the proposed mixtures and their potential relevance for underground mining support applications. The results showed that after 100 days, the 8% copper tailings mixture reached a compressive strength of 41.6 MPa, compared with 39.6 MPa for the conventional mixture. The addition of steel fibers produced a slight reduction in compressive strength and stiffness at early curing ages; however, comparable mechanical performance was achieved after extended curing. The observed improvements are primarily consistent with the filler effect and improved particle packing associated with the fine tailings fraction, although the underlying microstructural mechanisms were not directly evaluated in this study. Overall, the results provide preliminary evidence that copper tailings can be incorporated into laboratory-cast concrete mixtures with steel fibers, supporting their further evaluation for potential underground support applications.

## 1. Introduction

In recent years, there has been growing interest in the reuse of mining waste as raw material in construction applications, driven by the increasing demand for sustainable practices in the industry and the environmental liabilities associated with tailings deposits [1,2]. Chile, as one of the world’s major copper producers, generates large volumes of copper tailings annually, creating significant challenges in terms of their storage and long-term management [3]. It ranks third globally in the amount of tailings produced, after China and the United States [4]. The valorization of tailings as components of cement-based materials has been investigated with promising results, particularly regarding mechanical strength and durability when properly processed and proportioned [1,5,6,7]. Nevertheless, the variability in composition, granulometry, and potential chemical contaminants present in tailings introduces some technical uncertainties [5,6]. Interest in tailing recycling is also driven by the growing demand for low-carbon concrete, as reducing the use of conventional cementitious binders and increasing the incorporation of suitable waste-derived materials can contribute to lowering the environmental footprint of concrete [8,9]. Recent assessments have also shown that passive carbonation of concrete provides only a limited offset to the CO_2_ emissions associated with cement production, highlighting the importance of strategies that reduce emissions at the material production stage [10].

Underground excavations are subjected to complex geomechanical conditions associated with stress redistribution, fracturing, and progressive rock mass degradation. To maintain excavation stability, mining operations employ several ground support systems, including rock bolts, cable bolts, steel mesh, and shotcrete, either individually or in combination depending on the rock mass conditions [11,12,13,14,15]. Among these methods, shotcrete has become one of the most widely adopted support systems due to its rapid application, strong adhesion to rock surfaces, and ability to provide continuous confinement immediately after excavation [12,16].

The mechanical performance of shotcrete is governed by several factors, including the water-to-cement ratio, curing conditions, aggregate characteristics, and the use of chemical admixtures [17,18]. Compressive strength and elastic modulus are among the primary parameters used to evaluate its structural performance, while tensile strength and post-cracking behavior are particularly important for underground support applications subjected to dynamic loading [11,19]. The incorporation of steel fibers has become a common practice because it improves crack control, residual strength, toughness, and energy absorption capacity, transforming the brittle response of conventional shotcrete into a more ductile material [17,20,21,22,23]. Külikçi [24], Zhao et al. [25], Jeng et al. [26], and Banthia et al. [27] demonstrated that fiber type, geometry, and dosage strongly affect toughness, crack propagation, and energy absorption. However, Leung et al. [28] reported only minor differences in compressive strength between fiber-reinforced concrete and fiber-reinforced shotcrete, and Coviello et al. indicate that the incorporation of steel fibers had only a limited influence on compressive strength and elastic modulus, particularly at early curing ages [29].

Some studies have investigated the use of mineral tailings in shotcrete production [30,31,32]. The reuse of mine tailings as components of cementitious materials has received increasing attention as part of circular economy strategies within the mining industry [1,33]. Waste-derived cementitious materials are increasingly investigated not only for their contribution to circular economy strategies but also as a means for reducing clinker demand and the associated carbon footprint of concrete. Copper tailings commonly contain fine silicate-rich particles that can act as fillers and, depending on their mineralogical composition and processing history, may also contribute to secondary cementitious reactions [34,35,36,37,38,39,40]. Some studies have reported that partial replacement of natural aggregates or supplementary cementitious materials with mine tailings can maintain or even improve the mechanical performance and durability of concrete when appropriate replacement ratios are used [6,35]. However, their performance depends on factors such as particle size distribution, mineralogy, chemical composition, and curing conditions. Furthermore, concerns remain regarding the presence of sulfides and other potentially reactive minerals that may affect long-term durability if not properly characterized [1,35]. Extended curing periods have also been reported to play an important role in the strength development of tailings-based cementitious materials, particularly after 28 days, when slower hydration and secondary reactions continue contributing to the mechanical response [28,41,42].

Previous studies have demonstrated the potential of incorporating mine tailings as partial replacements for fine aggregate in shotcrete and have investigated their effects on mechanical and durability properties [30,32]. More recently, research specifically addressing copper tailings has reported promising results for their use as fine aggregate in shotcrete and cement-based mixtures, including mechanical performance, early-age behavior, durability, and environmental compatibility [6,43,44]. However, these studies have primarily considered copper tailings without steel-fiber reinforcement or have focused on conventional cast cementitious materials. Consequently, limited experimental information is available on the combined use of copper tailings and steel fibers, particularly regarding the evolution of compressive strength and elastic modulus over extended curing periods. This gap is relevant because the mechanical response of fiber-reinforced mixtures may differ from that of unreinforced tailings-based mixtures, while the effects of curing age remain important for assessing their potential use in underground mining support. Therefore, this study evaluates the mechanical performance of laboratory-cast concrete mixtures incorporating copper tailings as a partial replacement for natural fine aggregates to then compare the effect of adding copper tailings plus different steel fiber contents, focusing on the evolution of compressive strength and elastic modulus from 7 to 100 days.

## 2. Materials and Methods

### 2.1. Materials

The materials used in this study included cement, water, aggregates, copper tailings, and steel fibers:

- Cement: Type I Portland cement (Transex Plus, Santiago, Chile) was used as the binder in all mixtures.
- Aggregates: Natural sand was used as the aggregates in all mixtures (Santiago, Chile). In selected mixtures, a portion of the aggregates was replaced with copper tailings.
- Copper Tailings: Sourced from a Chilean copper mine, from an Iron Oxide–Copper–Gold (IOCG) deposit, the tailings originated from a hydrometallurgical process. Their fine granulometry was essential for homogeneous integration with other components.
- Water: Potable water complying with Chilean standard [45]. Water for concrete mixing was used.
- Steel Fibers: DRAMIX^®^ 45/50BL steel fibers (Prodalam, Santiago, Chile, 45/50, according to the manufacturer’s designation) had a nominal length of 50 mm and a diameter of 1.05 mm, corresponding to a calculated aspect ratio of approximately 48. These low-carbon, cold-drawn, circular-section fibers have a nominal tensile strength of 1115 MPa, and a Young’s modulus of 200 GPa.

### 2.2. Tailings Characterization

The copper tailings were characterized by the Laboratory of Rock Mechanics of University of Concepcion. The characterization aimed to determine their physical, chemical, and mineralogical properties to evaluate their suitability as partial replacement for fine aggregates for potential underground mining applications.

#### 2.2.1. Physical Characterization

The physical characterization included particle size distribution, density, water absorption, and shear strength tests. Particle size distribution was determined according to Chilean Standard NCh165 [46] using an electromagnetic sieve shaker (Matest, Treviolo, Italy YGM15418). Density and water absorption were measured following NCh1239. Direct shear tests were performed at normal stresses of 9.8, 19.6, 49, 98, and 147 kPa using a Matest ShearLab S276-10 apparatus, with a shear displacement rate of 0.2 mm/min. These analyses provided the main physical parameters to assess the suitability of the tailings as fine aggregate substitutes in concrete.

#### 2.2.2. Mineralogical and Chemical Characterization

The mineralogical composition was determined using automated mineralogy (QEMSCAN^®^), while the bulk chemical composition was obtained by wavelength dispersive X-ray fluorescence spectroscopy (XRF-WDS).

Automated Mineralogical Analysis (QEMSCAN^®^): The QEMSCAN^®^ 650 system, equipped with a Quanta 250 scanning electron microscope and two Bruker (Billerica, MA, USA) XFlash 6130 silicon drift detectors (nitrogen-free), was used for automated mineralogical mapping. Samples were prepared as polished briquettes containing both fine and coarse fractions of the tailings as received. The surface of each briquette was carbon-coated before analysis.

Measurements were conducted under the Particle Mineralogical Analysis (PMA) mode using the CuS-1 Specific Identification Protocol (SIP). Analyses were performed with a 5 µm point spacing, an acceleration voltage of 25 kV, and a beam current of 5 nA, under high vacuum (<5 × 10^−5^ Torr). The QEMSCAN images were acquired with an average working distance of 10 mm and a field size of 2000 µm (200× magnification). Calibration was performed using quartz (44), copper (130), and gold (232) for BSE intensity, and Cu (Lα and Kα) lines for spectrometer calibration.

The QEMSCAN^®^ analysis provided quantitative data on modal mineral abundance (% wt), mean grain size and textural associations, mineral liberation degree and intergrowth relationships, phase grouping based on chemical similarity and relevance for pozzolanic potential.

X-Ray Fluorescence (XRF): Bulk chemical composition was determined using a Rigaku ZSX Primus II wavelength dispersive spectrometer (Rh radiation, non-standardized mode). Dried samples were finely ground and pressed into 5 g pellets under high pressure to produce smooth, homogeneous surfaces. The pellets were analyzed under vacuum conditions to obtain semi-quantitative oxide concentrations.

This analysis allowed identification of major oxides such as SiO_2_, Fe_2_O_3_, Al_2_O_3_, CaO, K_2_O, and MgO, which are key indicators of pozzolanic reactivity and mechanical performance potential for cementitious applications.

### 2.3. Mix Design and Procedure

The experimental methodology led to the fabrication of 48 cubic specimens (102 mm per side), distributed across four configurations. Each configuration was tested at four curing times (7, 14, 28, and 100 days), with three replicates per group to ensure statistical representativeness. Four different mixtures were prepared:

- Control concrete: Traditional composition without tailings or fibers.
- Tailings-based concrete: Replacement of 8% of the total mixture with copper tailings.
- Tailings-based concrete + 0.4% Steel Fibers: Tailings mixture with 0.4% fiber reinforcement by mass of the base mixture, added as an additional constituent.
- Tailings-based concrete + 1.2% Steel Fibers: Tailings mixture with 1.2% fiber reinforcement by mass of the base mixture, added as an additional constituent.

In Table 1 the proportions of cement, water, natural aggregates, and copper tailings are expressed as percentages of the base mixture mass. Copper tailings replaced 8.0 wt.% of the total base mixture mass, with an equivalent reduction in natural fine aggregate. Steel fibers were added additionally at 0.4% and 1.2% of the base mixture mass and did not replace any other constituent; therefore, the total mass of the fiber-reinforced mixtures exceeds 100% of the base mixture mass by the corresponding fiber dosage.

All mixes were prepared in a 20 L paddle-type mechanical mixer following a standardized protocol to ensure uniformity:

1. Initial hydration: A total of 70% of total mixing water was poured into the container.
2. Partial aggregate addition: A total of 50% of the aggregate fraction (72.4% for base mix, 64.4% for tailings mix) was added.
3. Cement incorporation: All cement (17.5%) was added and mixed for 7 min at a constant speed (30 rpm).
4. Remaining aggregates: The remaining 50% of aggregates were added and mixed for another 7 min.
5. Remaining water: The final 30% of mixing water was added, followed by 7 min of mixing.
6. Fiber addition: For reinforced mixtures, DRAMIX^®^ 45/50BL fibers were added (0.4% or 1.2% by mass of the base mixture, added as an additional constituent) and mixed for an additional 7 min to prevent clumping.

The aggregates and copper tailings were oven-dried at 105 °C for 48 h before mixing. No correction of the mixing water for aggregate or tailings absorption was applied. The resulting mixture was placed in lubricated steel molds (102 mm cubes), compacted manually with a tamping rod, and allowed to rest at ambient temperature (15–25 °C) for 24 h to complete initial setting, following ASTM C192/C192M [47].

After 24 h, the specimens were demolded. A total of 48 specimens were prepared for the mechanical testing program, corresponding to four mixtures, four curing ages, and three replicate specimens for each mixture–curing age combination (4 × 4 × 3 = 48). Compressive strength and elastic modulus were determined using the same specimens.

Fresh density was also determined for the control and tailings mixtures. The measured wet densities were 2200 and 2160 kg/m^3^ for the control and tailings mixtures, respectively. The corresponding dry densities were 1970 and 2010 kg/m^3^. These measurements were used to characterize the fresh and hardened mixture densities under the laboratory conditions adopted in this study.

### 2.4. Mechanical Testing

Tests were conducted using a servo-hydraulic universal testing machine (Advantest 9, Controls, Milan, Italy) capable of static and dynamic load applications. Tests followed EN-12390-3 [45] using 102 mm cubic specimens. Stress was computed as the ratio of applied force to the cross-sectional area. The peak stress value was recorded as UCS. The elastic modulus was determined using a procedure adapted from ASTM C469/C469M [48] for the laboratory-cast 102 mm cubic specimens used in this study. The elastic modulus was determined using an axial displacement measurement obtained with an LVDT mounted directly on the specimen. The gauge length was 50 mm, and the loading rate was 0.25 MPa/s. The axial strain was calculated as the measured displacement divided by the corresponding gauge length. The modulus was obtained from the slope of the stress–strain response over the specified stress range. The modulus was derived from the linear portion of the stress–strain curve, up to 40% of the maximum load. Mean values, standard deviations, and coefficients of variation were computed for each configuration. Trends in strength and stiffness were analyzed across curing durations and between formulations. The impact of tailings and steel fibers was evaluated using comparative plots and statistical indicators.

## 3. Tailings Characterization Results

### 3.1. Physical Characterization of Copper Tailings

**Figure 1:**
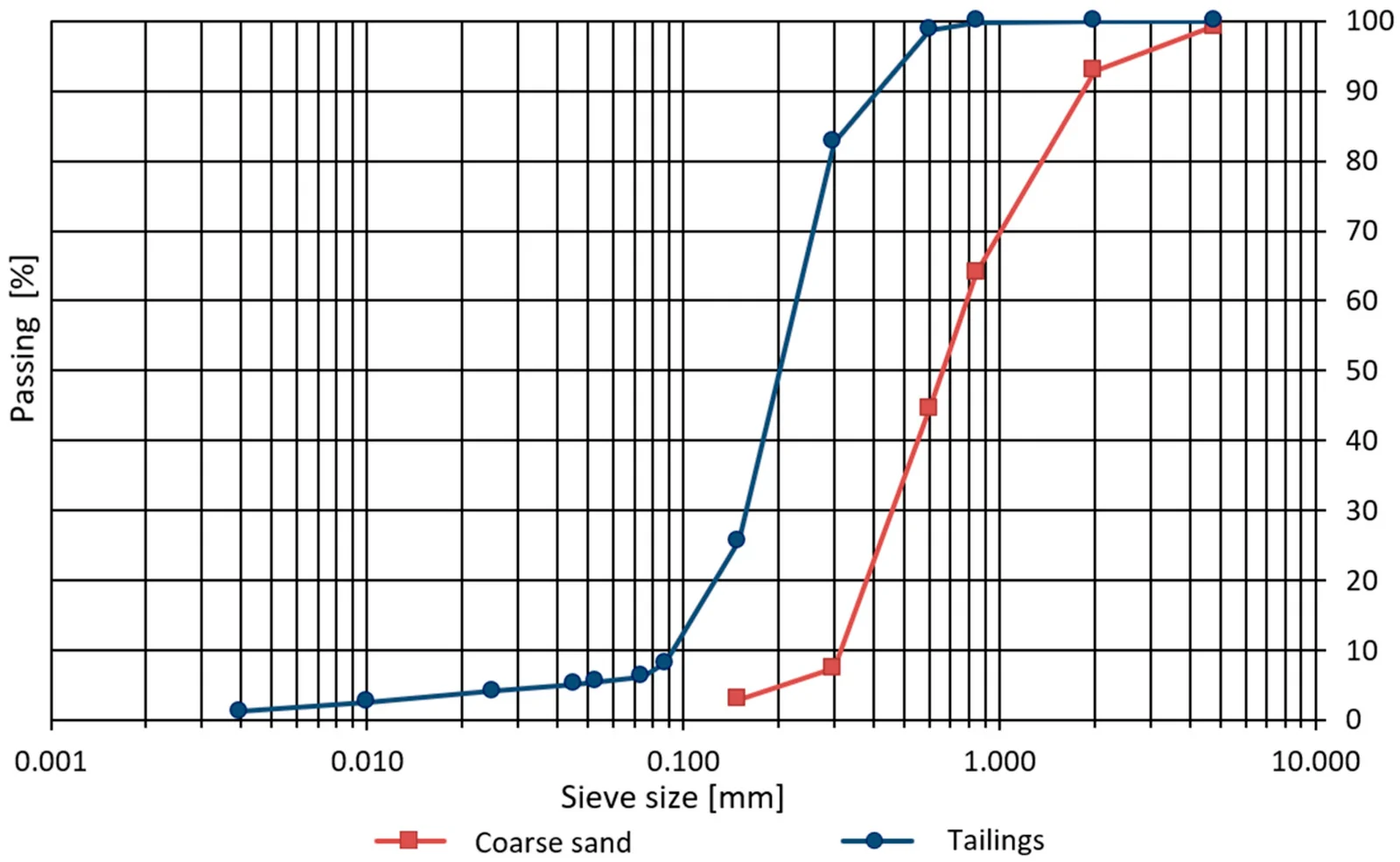
Particle size distribution curve of copper tailings and coarse sand.

The particle size analysis revealed that the copper tailings are composed predominantly of fine particles, with a characteristic D_80_ of approximately 290 µm and D_10_ of 93 µm. The particle-size distribution of the tailings differs markedly from that of the natural coarse sand, showing a substantially finer particle-size range (Figure 1). The relatively low uniformity coefficient (Cu = 2.45) indicates a poorly graded particle-size distribution. Particle-size distribution was determined by sieve analysis for particles larger than 75 μm, while the fraction below 75 μm was characterized using laser diffraction.

These parameters suggest that the partial substitution of natural sand by tailings could improve the packing density of the fine fraction. The specific gravity and water absorption results are summarized in Table 2. The tailings exhibited a higher dry and saturated surface-dry density (2.81 and 2.88 t/m^3^, respectively) compared to natural coarse sand (2.66 and 2.71 t/m^3^).

However, the tailings also showed greater water absorption (2.32%) than sand (1.84%), which could negatively influence the workability of fresh mixtures. Therefore, adjustments to the water-to-cement ratio or the use of plasticizing admixtures may be necessary to ensure adequate rheological behavior.

**Figure 2:**
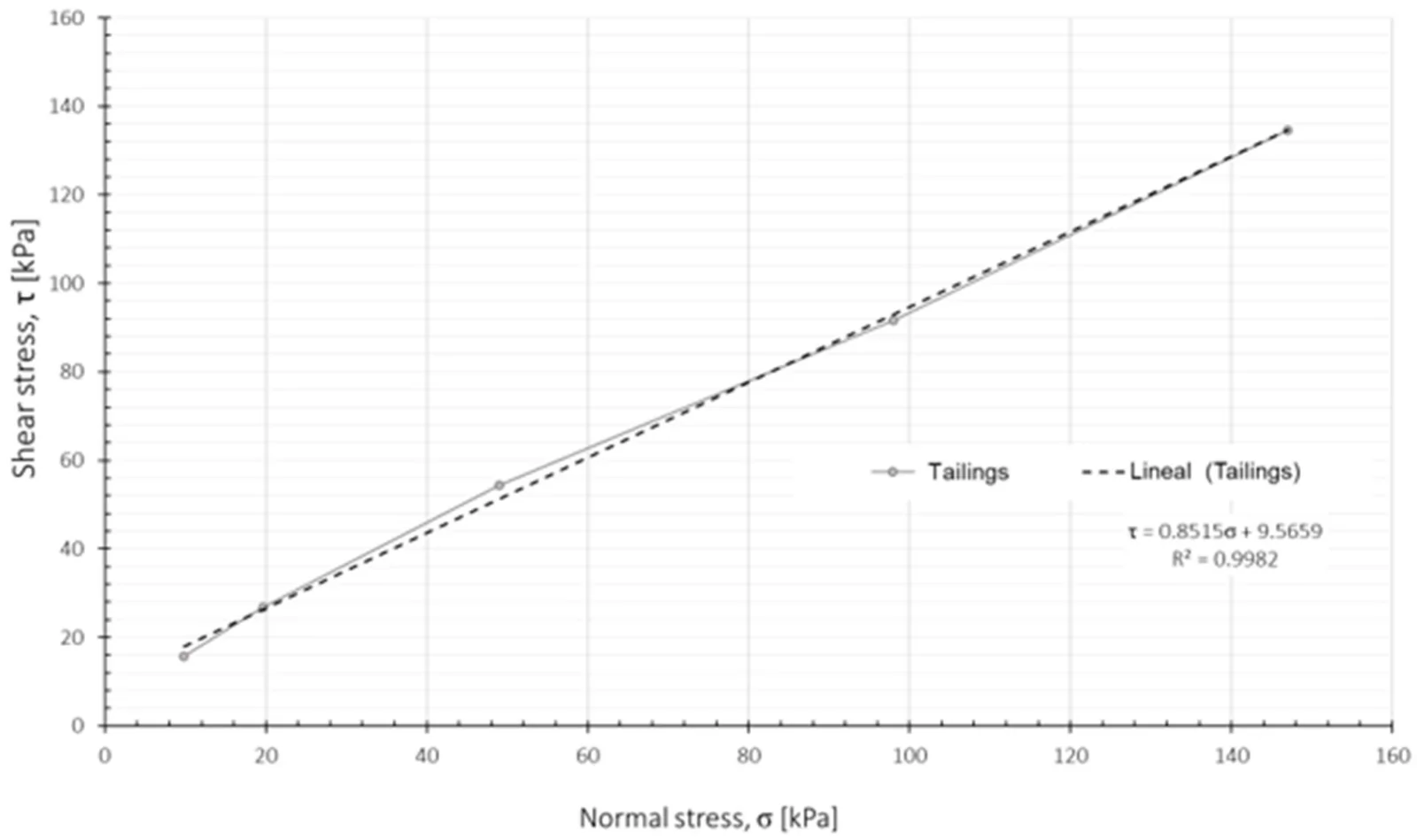
Shear stress vs. Normal stress for copper tailings.

Direct shear tests (Figure 2) indicated a low cohesion (9.6 kPa) but a relatively high internal friction angle (48.8°), with a coefficient of determination (R^2^ = 0.998) for the linear fitting of shear stress versus normal stress. The relatively high friction angle confirms the granular nature of the tailings and indicates that they possess adequate mechanical stability for use as fine aggregate replacement.

### 3.2. Mineralogical and Chemical Characterization of Copper Tailings

The automated mineralogical analysis (Table 3) revealed that the copper tailings are predominantly composed of K-feldspar (18.7 wt.%), quartz (17.6 wt.%), chlorite (16.5 wt.%), and iron oxides/hydroxides (15.1 wt.%), with smaller proportions of plagioclase, biotite, pyroxene-amphibole, and pyrite. Together, these major mineral phases account for more than 90 wt.% of the total mineralogical composition, indicating that the tailings are dominated by silicate-rich minerals.

The predominance of quartz and feldspars, together with the fine particle size of the tailings, suggests that the material may contribute to improving particle packing within the cementitious matrix. In addition, the predominance of aluminosilicate minerals is consistent with compositions previously reported for copper tailings used in cementitious materials and may contribute to the long-term mechanical performance through filler effects and possible secondary cementitious reactions. The low abundance of sulfide minerals (<3 wt.%), mainly represented by pyrite, also reduces potential concerns associated with sulfide oxidation in cement-based applications.

The potential sulfate- and sulfide-related durability risks associated with the incorporation of copper tailings were not specifically evaluated in the present study. Although the measured chemical and mineralogical composition provides useful information regarding the presence of sulfur-bearing phases, no expansion, length-change, or long-term durability tests were conducted. Therefore, the potential for sulfate-related expansion or other sulfur-related degradation mechanisms cannot be assessed from the present experimental program. Dedicated long-term durability and leaching investigations should be considered in future studies, particularly for applications involving prolonged exposure to mine water. Previous studies have highlighted durability and acid-attack considerations for copper-tailings-based cementitious materials [6]. However, the present study focused on mechanical performance and did not include dedicated durability or leaching assessments.

The average grain size of the identified mineral phases ranged from approximately 8 to 31 µm (Table 3), indicating that most particles occur within the fine fraction. This fine mineralogy is considered advantageous for achieving a more homogeneous particle distribution within cementitious mixtures and supports the evaluation of these tailings as a partial replacement for natural fine aggregates.

The mineralogical composition indicates that the tailings are primarily composed of silicate-rich minerals, which are commonly found in natural aggregates and have been widely investigated for cementitious applications. To complement the mineralogical characterization, the bulk chemical composition of the tailings was determined by X-ray fluorescence (XRF; Table 4), allowing the identification of the principal oxides that may influence the behavior of the material when incorporated into cementitious mixtures.

The XRF analysis showed that the tailings are primarily composed of SiO_2_ (45.35 wt.%), Fe_2_O_3_ (24.12 wt.%), and Al_2_O_3_ (12.53 wt.%), which together account for more than 80 wt.% of the total oxide composition. Smaller amounts of MgO, K_2_O, and CaO were also identified, together with minor concentrations of SO_3_ and trace elements.

The predominance of silica and alumina is consistent with the mineralogical composition identified by QEMSCAN and with the characteristics commonly reported for copper tailings used in cementitious materials. These oxides, together with the fine particle size of the tailings, may contribute to improved particle packing and, under favorable conditions, secondary cementitious reactions [49,50]. However, the present study did not directly evaluate these mechanisms, and therefore they are proposed only as possible contributors to the mechanical behavior discussed in the following section.

Overall, the physical, mineralogical, and chemical characterization indicates that the copper tailings possess suitable particle size distribution and a predominantly silicate-rich composition, supporting their evaluation as a partial replacement for natural fine aggregates in concrete mixtures. The following section examines whether these characteristics translate into improved mechanical performance.

## 4. Mechanical Performance and Discussions

Subsequent analysis includes the evaluation of uniaxial compressive strength and elastic modulus, examining their temporal evolution and the potential synergistic effects of tailings and fiber reinforcement. The consolidated dataset (Table 5) presents average values for strength and stiffness (in MPa), along with standard deviation (SD) and coefficient of variation (CV%) for each mixture and curing time. These properties were obtained from the data collected during mechanical testing, which involved the measurement of axial force and displacement using the servo-hydraulic system Advantest 9 by Controls. The compressive stress was calculated from the applied force and contact surface area, allowing for the determination of the peak compressive strength and stiffness of each specimen.

Overall, both the compressive strength and elastic modulus increased with curing time for all mixtures, confirming the continued development of mechanical properties with age. Pronounced increases were indeed observed at 7 days; however, the mechanical properties measured at this age, particularly strength and elastic modulus, can exhibit relatively high experimental variability (e.g., [51,52,53]). This is reflected in the relatively high coefficients of variation observed for the tailings concrete at 7 days, reaching 10% for compressive strength and 18% for elastic modulus. At later ages, the tailings-only concrete continued to gain strength, increasing from 38.1 MPa at 28 days to 41.6 MPa at 100 days, while its elastic modulus increased from 11.2 to 13.6 GPa. Interestingly, the tailings-only concrete also exhibited a relatively high coefficient of variation at 100 days, reaching 11% for compressive strength and 18% for elastic modulus. Therefore, although the tailings-only concrete exhibited approximately 5% higher compressive strength than the control concrete at 100 days (41.6 versus 39.6 MPa), this difference should be interpreted with caution given the observed experimental variability. This variability may be related to the delayed strength development observed in some tailings-based cementitious mixtures, which can exhibit continued strength development at later curing ages [35,54,55,56].

The incorporation of steel fibers generally resulted in lower compressive strength and elastic modulus than the plain tailings concrete, particularly at early ages, although both properties continued to increase with curing time. The mixtures containing 0.4% and 1.2% steel fibers reached compressive strengths of 37.8 and 38.2 MPa, respectively, at 100 days, compared with 41.6 MPa for the tailings concrete without fibers. From an engineering perspective, the relevance of this result, therefore, lies primarily in demonstrating that partial replacement of natural fine aggregate with copper tailings can maintain mechanical performance comparable to the conventional mixture, rather than in the magnitude of the observed strength increase itself.

### 4.1. Compressive Strength

**Figure 3:**
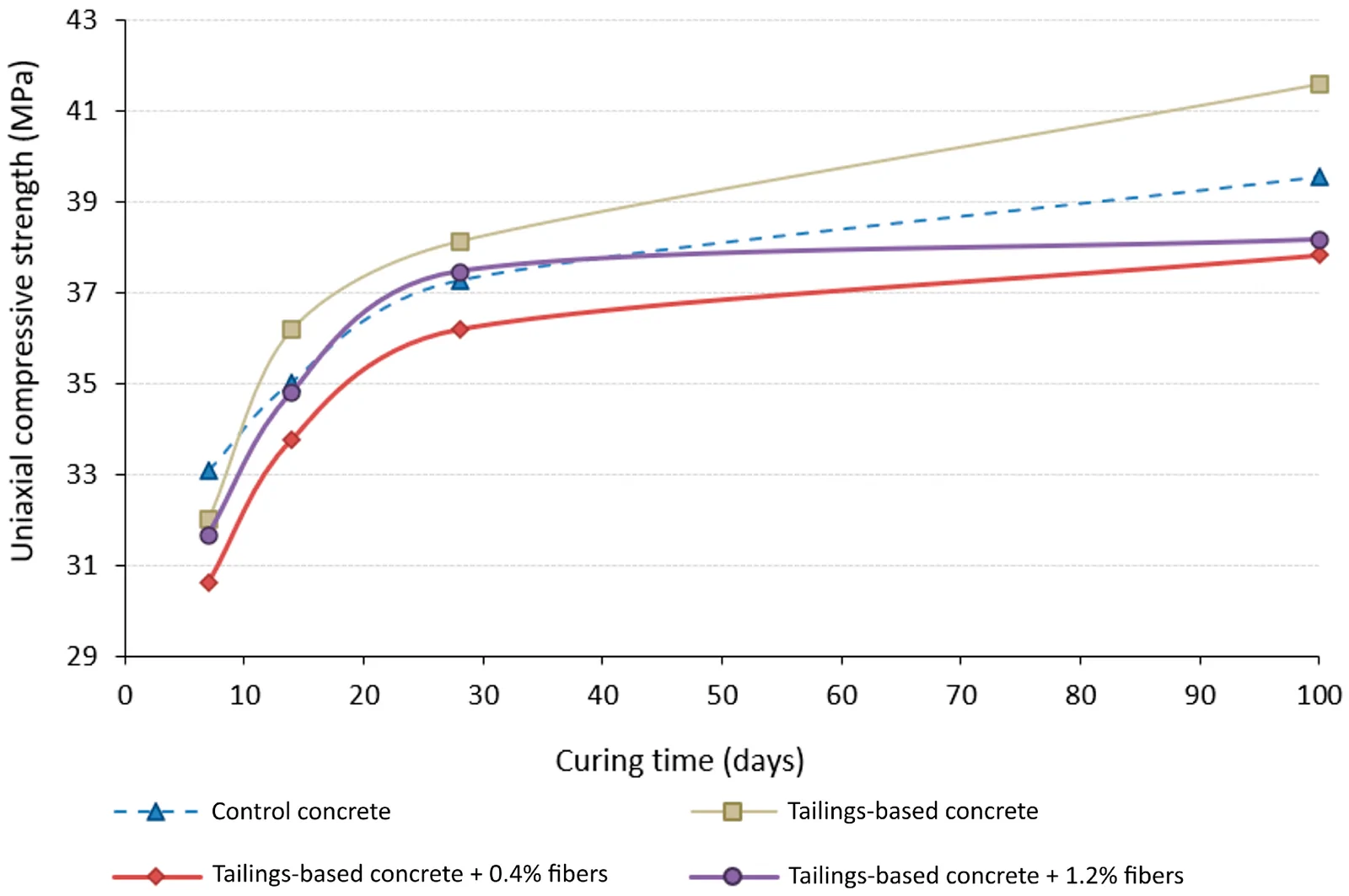
Comparison of uniaxial compressive strength by sample type in relation to curing time.

Figure 3 illustrates the relationship between mechanical performance, curing time, and mixture composition, highlighting key behavioral trends. The analysis of uniaxial compressive strength evolution across different curing times revealed distinct patterns influenced by the incorporation of copper tailings and steel fibers. At 7 days of curing, conventional concrete exhibited the highest strength, followed closely by the mix containing tailings. The fiber-reinforced samples, particularly the mixture with 0.4% fiber, showed a slight reduction in compressive strength. This behavior may be associated with changes in mixture homogeneity and fresh-state compactability caused by the incorporation of steel fibers. Fiber addition can modify the workability of concrete and, depending on fiber dosage and mixture characteristics, may contribute to local heterogeneity, entrapped voids, or less effective compaction [57]. By 14 days, the concrete containing tailings slightly surpassed the conventional mix in compressive strength. This behavior may be associated with improved particle packing and potentially with secondary cementitious reactions reported for tailings-based cementitious materials; however, these mechanisms were not directly evaluated in the present study. The steel-fiber-reinforced samples remained slightly lower in strength and showed greater variability, particularly for the 0.4% fiber content.

At 28 days, the strength trend continued, with tailings-enhanced concrete outperforming the conventional mix. Fiber-reinforced samples displayed similar strength values, though the group with 1.2% fiber content showed higher variability, likely due to the challenges in achieving uniform fiber dispersion. At 100 days, the tailings-only concrete achieved the highest compressive strength (41.6 MPa), followed by the conventional concrete (39.6 MPa), whereas the mixtures containing 0.4% and 1.2% steel fibers reached 37.8 and 38.2 MPa, respectively. These results suggest that the incorporation of steel fibers had a more pronounced effect on compressive strength during the early curing period, although the strength of all fiber-reinforced mixtures continued to increase with curing time.

The results showed a nonlinear increase with curing time, with more pronounced gains at early ages and progressively smaller increases at later ages. This behavior is consistent with cementitious materials, where cement hydration is a continuous process that extends well beyond the initial 28 days [19]. During the first 28 days, strength increases significantly due to the rapid formation of calcium silicate hydrates (C-S-H), which densify the cementitious matrix. After this period, the rate of strength gain slows down substantially. This deceleration is explained by the progressive depletion of reactive compounds, such as calcium and silicon oxides, which limit the formation of new hydration products [58]. Despite the reduced rate, residual hydration continues even at 100 days, contributing to marginal final strength improvements.

The observed behavior is primarily consistent with the filler effect and improved particle packing associated with the fine tailings fraction. Previous studies have reported secondary cementitious or pozzolanic activity in selected mine tailings under specific mineralogical or activation conditions [35,37,38,39]; however, such reactions were not directly evaluated in the present study and, therefore, cannot be invoked as a confirmed mechanism for the observed strength development.

The fine granulometry of the tailings facilitates the filling of interstitial spaces within the cement-aggregate mixture, leading to improved compaction and reduced matrix porosity. This reduction in porosity is key, as it enhances the uniaxial compressive strength and can simultaneously decrease permeability. Minimizing permeability is essential for ensuring the long-term durability of concrete in aggressive environments, such as those encountered in some underground mining [11].

### 4.2. Elastic Modulus

**Figure 4:**
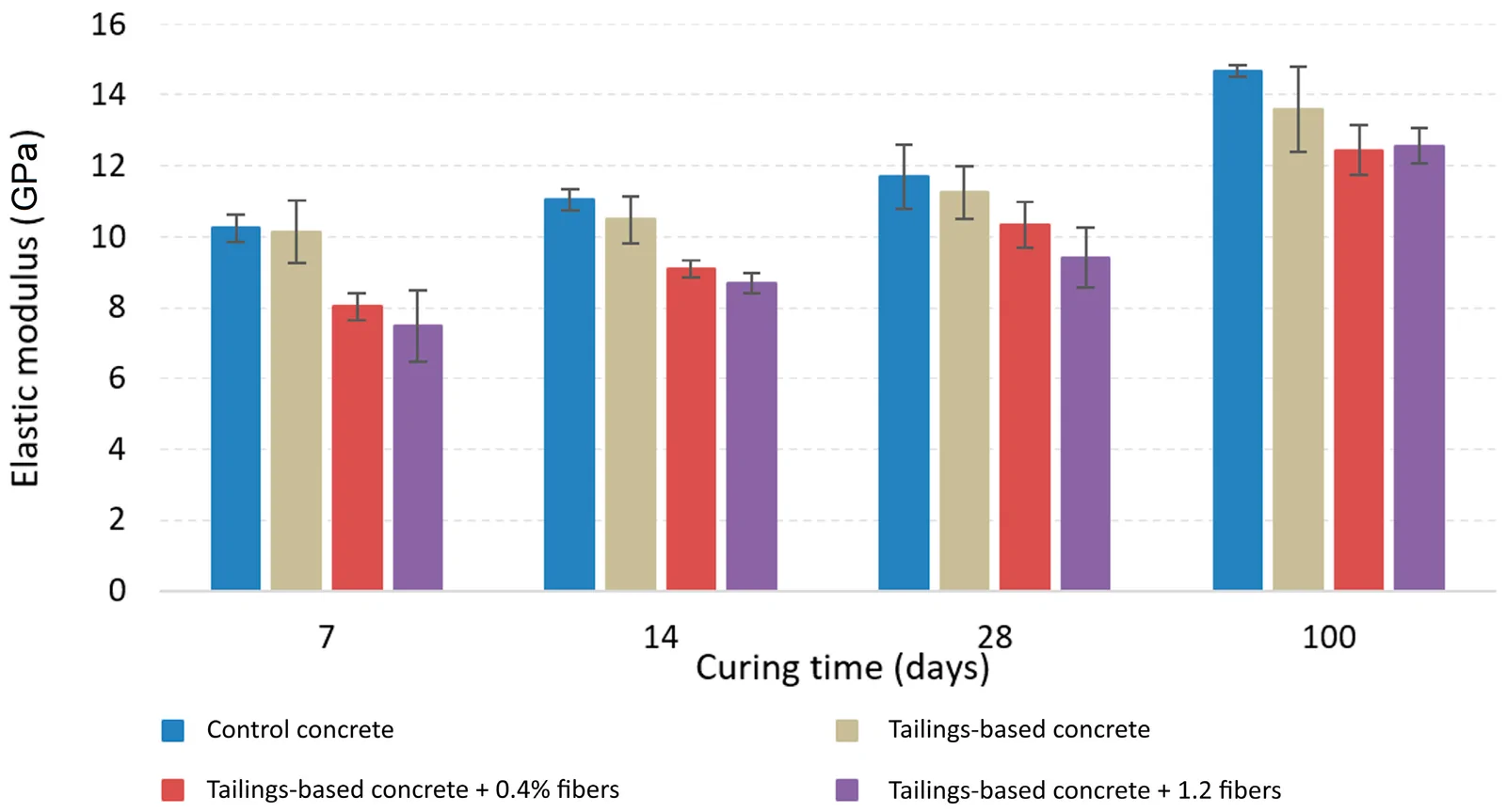
Evolution of the elastic modulus of concrete mixtures as a function of curing time.

In Figure 4, the elastic modulus exhibited a progressive increase with curing time for all mixtures. The measured values were relatively low compared with those estimated from conventional code-based empirical relationships for concrete of similar compressive strength. Nevertheless, the results showed a consistent age-dependent trend, with the modulus increasing from 10.2 to 14.7 GPa for the control concrete and from 10.1 to 13.6 GPa for the tailings-only concrete between 7 and 100 days. The relatively low modulus values may be associated with the specific composition and aggregate characteristics of the mixtures investigated, although the present study was not designed to isolate the contribution of individual constituents to the measured stiffness. Therefore, the modulus results are primarily interpreted in terms of relative differences and evolution with curing age rather than as absolute values for structural design.

At 100 days, the measured modulus values increased for all mixtures, with the tailings-based mixtures reaching 13.6 GPa without fibers and approximately 12.5–12.6 GPa with steel fibers, compared with 14.7 GPa for the control mixture. Thus, although the absolute stiffness values remained below conventional code-based estimates, the tailings-only based mixtures exhibited a substantial increase in stiffness with curing age and approached the stiffness of the control mixture under the adopted testing conditions.

The addition of fibers produced a nonlinear effect on the overall stiffness of the material. At higher fiber content (1.2%), a slight reduction in the measured modulus was observed. This decrease may be associated with increased heterogeneity and possible void formation within the matrix, potentially resulting from fiber clustering and difficulties in achieving optimal compaction at elevated fiber dosages. These discontinuities can reduce the efficiency of the load transfer mechanism between the matrix and the steel fibers during the initial stages of loading.

The long-term results indicate that the measured stiffness of the steel fiber-reinforced tailings mixtures increased with curing age and tended to approach that of the reference mixture. This trend may be related to continued development of the cementitious matrix and improved load transfer, although the underlying interfacial mechanisms were not directly characterized.

### 4.3. Overall Mechanical Behaviour

The combined evaluation of compressive strength and elastic modulus indicates that the incorporation of copper tailings did not adversely affect the mechanical performance of the concrete mixtures. On the contrary, the tailings-based mixtures exhibited comparable or slightly higher values than the reference mixture after extended curing, demonstrating that a moderate replacement of natural fine aggregates can be achieved without compromising the structural response of the material. Similar long-term improvements have been reported in previous studies on copper tailings incorporated into cementitious materials, where the fine particle size of the tailings contributed to improved matrix densification and mechanical performance [5,6]. The similar evolution observed for both compressive strength and elastic modulus suggests that the mechanical response was primarily governed by the progressive development of the cementitious matrix during curing. Although the present study did not directly investigate the underlying microstructural mechanisms, the results are consistent with the combined influence of improved particle packing associated with the fine tailings fraction and possible secondary cementitious reactions reported for copper tailings in the literature. The continuous increase in both properties between 28 and 100 days further highlights the importance of extended curing when evaluating alternative cementitious materials.

Regarding fiber reinforcement, the results indicate that the incorporation of steel fibers had only a limited influence on compressive strength and elastic modulus, particularly at early curing ages. This behavior is expected because the primary contribution of steel fibers is generally associated with post-cracking performance, toughness, and energy absorption rather than compressive loading. Consequently, the similar compressive properties obtained for the fiber-reinforced and unreinforced mixtures suggest that the inclusion of copper tailings can be implemented without negatively affecting the conventional mechanical properties of fiber-reinforced concrete.

It should be noted that the specimens investigated in this study were laboratory-cast and were not produced through an actual shotcrete spraying process. Therefore, the present results characterize the mechanical behavior of the proposed mixtures under controlled laboratory casting conditions and should not be interpreted as a direct assessment of sprayed shotcrete performance. In particular, the effects of spraying on fiber orientation, compaction, rebound, and the resulting mechanical properties were not evaluated. Further experimental work using an actual shotcrete spraying setup is required to assess the field applicability of these mixtures.

An additional limitation concerns the specimen dimensions relative to the steel fiber length. The cubic specimens had an edge length of 102 mm, whereas the steel fibers had a nominal length of 50 mm. Therefore, the specimen dimension-to-fiber-length ratio was approximately 2.0, which may introduce preferential fiber alignment near the mold boundaries and make the measured fiber-related response dependent on specimen geometry. Furthermore, the manual rodding used for compaction may have contributed to fiber orientation. Consequently, the mechanical results obtained for the fiber-reinforced mixtures should be interpreted with caution, particularly with respect to fiber-related effects, and may not be directly representative of larger specimens or sprayed shotcrete. Further testing using specimen dimensions appropriate for the fiber length and controlled casting or spraying procedures would be required to isolate the intrinsic effect of fiber content.

In this study the copper tailings were used as a partial replacement for natural fine aggregates while the cement content was kept constant. Therefore, the observed mechanical response is more directly related to the physical effects of the fine tailings fraction, particularly particle packing and matrix densification, than to a demonstrated pozzolanic contribution. From an engineering perspective, these findings demonstrate that replacing 8% of the natural fine aggregates with copper tailings can represents a viable alternative for shotcrete applications in underground mining. The proposed mixtures achieved mechanical properties comparable to those of conventional shotcrete while simultaneously promoting the reutilization of mining waste, supporting more sustainable construction practices within the framework of the circular economy.

## 5. Conclusions

This study demonstrates the potential of incorporating copper tailings and steel fibers into laboratory-cast concrete mixtures. The main conclusions are the following:

- The copper tailings used exhibited predominantly fine particle-size characteristics. This composition may have contributed to improved particle packing and, matrix densification. The resulting effects are consistent with the observed long-term strength development, although microstructural mechanisms were not directly characterized in this study. At 100 days, the tailings-only mixture reached 41.6 MPa, compared with 39.6 MPa for the control mixture; this difference should be interpreted with caution given the observed experimental variability. Although secondary cementitious reactions have been reported for some tailings-based systems, their occurrence and contribution to strength development were not evaluated in the present study.
- A similar age-dependent trend was observed for the elastic modulus. Although the measured stiffness values were lower than conventional code-based estimates for concrete with similar compressive strength, all mixtures exhibited increasing stiffness with curing age. The tailings-only concrete increased from 10.1 GPa at 7 days to 13.6 GPa at 100 days, while the control increased from 10.2 to 14.7 GPa. The results, therefore, provide useful information on the relative evolution of stiffness among the investigated mixtures, while further standardized testing would be required before using these values for structural design.
- The tailings-based fiber-reinforced concrete developed in this study achieved mechanical performance comparable to conventional mixtures while contributing to improved sustainability and supporting the integration of circular economy principles in mining operations.

## Figures and Tables

**Table 1:** Mixture proportions of the laboratory-cast concrete mixtures.

Mix	Cement wt.%	Water wt.%	Aggregate wt.%	Copper Tailings wt.%	Steel Fiber wt.%
Control concrete	17.5	10.1	72.4	0	0
Tailings-based concrete	17.5	10.1	64.4	8	0
Tailings-based concrete with 0.4% fiber	17.5	10.1	64.4	8	0.4 *
Tailings-based concrete with 1.2% fiber	17.5	10.1	64.4	8	1.2 *

* Steel fibers were added additionally at 0.4% and 1.2% by mass of the base mixture and did not replace any other constituent.

**Table 2:** Density and water absorption for each material.

Material	Saturated Surface-Dry Density g/cc	Dry Density g/cc	Apparent Particle Density g/cc	Water Absorption %
Coarse sand	2.71	2.66	2.80	1.84
Copper tailings	2.88	2.81	3.01	2.32

**Table 3:** Weight percentages of main mineral phases identified in the copper tailings.

Sample	Weight Percentage [%]	Average Mineral Size [µm]
K-Feldespar	18.68	23.4
Quartz	17.65	29.0
Chlorite	16.52	19.5
Oxides–Hydroxides Fe	15.08	23.4
Feldspars (Plagioclase)	10.31	24.8
Biotite	8.34	11.2
Pyroxene-Amphibole	3.58	17.9
Pyrite	2.42	21.0
Others	2.27	7.8
Carbonates (Ca-Fe-Mg)	1.32	21.5
Epidote	1.31	8.5

**Table 4:** Semi-quantitative whole-rock chemical composition determined by X-ray fluorescence (XRF).

Elements	Weight Percentage [%]	Elements	Weight Percentage [%]
F	0.08	Fe2O3	24.12
Na2O	1.94	Co2O3	*
MgO	5.46	NiO	*
Al2O3	12.53	CuO	0.06
SiO2	45.35	ZnO	*
P2O5	0.32	Ga2O3	*
SO3	2.36	As2O3	*
Cl	0.09	Rb2O	*
K2O	3.58	SrO	*
CaO	3.16	Y2O3	*
TiO2	0.54	ZrO2	*
V2O5	0.03	BaO	0.09
Cr2O3	0.02	WO3	0.03
MnO	0.16	PbO	-

* indicates trace amounts.

**Table 5:** Results of uniaxial compressive strength and deformation modules by sample type and curing time.

Sample Type	Curing Time (Days)	Uniaxial Compressive Strength (MPa)			Modulus of Elasticity (GPa)		
		Value	SD	CV (%)	Value	SD	CV (%)
Control concrete	7	33.1	1.80	5%	10.2	0.76	7%
Control concrete	14	35.0	0.85	2%	11.0	0.63	6%
Control concrete	28	37.3	0.57	2%	11.7	1.84	16%
Control concrete	100	39.6	3.32	8%	14.7	0.29	2%
Tailings-based concrete	7	32.0	3.22	10%	10.1	1.78	18%
Tailings-based concrete	14	36.2	0.82	2%	10.5	1.34	13%
Tailings-based concrete	28	38.1	1.08	3%	11.2	1.49	13%
Tailings-based concrete	100	41.6	4.51	11%	13.6	2.42	18%
Tailings-based concrete + 0.4% Steel Fibers	7	30.6	1.93	6%	8.0	0.76	9%
Tailings-based concrete + 0.4% Steel Fibers	14	33.8	1.76	5%	9.1	0.49	5%
Tailings-based concrete + 0.4% Steel Fibers	28	36.2	0.53	1%	10.3	1.32	13%
Tailings-based concrete + 0.4% Steel Fibers	100	37.8	1.53	4%	12.4	1.40	11%
Tailings-based concrete + 1.2% Steel Fibers	7	31.7	1.06	3%	7.5	2.03	27%
Tailings-based concrete + 1.2% Steel Fibers	14	34.8	0.66	2%	8.7	0.57	7%
Tailings-based concrete + 1.2% Steel Fibers	28	36.6	3.82	10%	9.4	1.68	18%
Tailings-based concrete + 1.2% Steel Fibers	100	38.2	2.25	4%	12.6	1.03	8%

## Data Availability

The original contributions presented in this study are included in the article. Further inquiries can be directed to the corresponding authors.
